# Ferulic Acid Derivatives and Avenanthramides Modulate Endothelial Function through Maintenance of Nitric Oxide Balance in HUVEC Cells

**DOI:** 10.3390/nu13062026

**Published:** 2021-06-12

**Authors:** Gabriele Serreli, Melanie Le Sayec, Estelle Thou, Camille Lacour, Camilla Diotallevi, Misbah Arshad Dhunna, Monica Deiana, Jeremy P. E. Spencer, Giulia Corona

**Affiliations:** 1 Health Sciences Research Centre, Life Sciences Department, Whitelands College, University of Roehampton, London SW15 4JD, UK; gabriele.serreli@unica.it (G.S.); melanie.le_sayec@kcl.ac.uk (M.L.S.); estelle.thou@roehampton.ac.uk (E.T.); camille.lacour@roehampton.ac.uk (C.L.); camilla.diotallevi@roehampton.ac.uk (C.D.); dhunnam@roehampton.ac.uk (M.A.D.); 2 Department of Biomedical Sciences, University of Cagliari, Cittadella Universitaria, SS 554, km 4.5, 09042 Monserrato, Italy; mdeiana@unica.it; 3 Molecular Nutrition Group, Food and Nutritional Sciences Department, University of Reading, Reading RG6 6AP, UK; j.p.e.spencer@reading.ac.uk

**Keywords:** ferulic acid, avenanthramides, bioactive, vascular function, superoxide, nitric oxide, endothelial cells, eNOS

## Abstract

Wholegrain oats contain a variety of phenolic compounds thought to help maintain healthy vascular function, through the maintenance of local levels of the vasodilator nitric oxide (NO). Thus, the full molecular mechanisms involved are not yet clear. With this work we aim to understand the possible cellular mechanisms by which avenanthramides and ferulic acid derivatives, present in oats, may help maintain a healthy vascular function through the modulation of the NO pathway. Primary Human Umbilical Vein Endothelial Cells (HUVEC) were exposed to ferulic acid, isoferulic acid, hydroferulic acid, ferulic acid 4-*O*-glucuronide, isoferulic acid 3-*O*-sulfate, dihydroferulic acid 4-*O*-glucuronide, avenanthramide A, avenanthramide B and avenanthramide C (1 μM) or vehicle (methanol) for 24 h. Apocynin and Nω-Nitro-L-arginine (L-NNA) were additionally included as controls. NO and cyclic GMP (cGMP) levels, superoxide production and the activation of the Akt1/eNOS pathway were assessed. The statistical analysis was performed using one-way ANOVA followed by a Tukey post-hoc t-test. Apocynin and all phenolic compounds increased NO levels in HUVEC cells (increased DAF2-DA fluorescence and cGMP), and significantly reduced superoxide levels. Protein expression results highlighted an increase in the Akt1 activation state, and increased eNOS expression. Overall, our results indicated that the glucuronide metabolites do not enhance NO production through the Akt1/eNOS pathway, thus all compounds tested are able to reduce NO degradation through reduced superoxide formation.

## 1. Introduction

Oats are a widely consumed wholegrain, and their consumption is consistently associated with a lower risk of chronic diseases, especially cardiovascular diseases [1]. The cardiovascular benefits of regular oat consumption are predominantly attributed to the fiber component [2], thus oats are extremely rich in other bioactives [3], including ferulic acid (FA), a phenolic acid present in all wholegrains [4], and avenanthramides (AVN), which are unique to oats [5,6]. The impact of oats intake on human endothelial function is therefore likely to be the result of the combined effects of both fiber and phenolic acids found within the wholegrain [7,8,9]. After ingestion, soluble forms of FA can be absorbed in the upper part of the gastrointestinal tract [10,11,12]. FA can be further metabolized in the colon into a number of additional forms [13,14], including isoferulic acid (IFA), dihydroferulic acid (DHFA) and further bio-transformed in the gut and in the liver to produce their -glucuronide and -sulfate conjugated forms [15,16,17]. AVN are also shown to be bioavailable after oat consumption [18,19], with AVN-B having the slowest elimination rate and the longest half-life compared to AVN-A and AVN-C [20]. Overall, the available literature shows that AVN, FA and FA metabolic derivatives are bioavailable forms present in the circulation after oat consumption [17,19,20,21]. Several animal studies indicated that FA can play a role in the maintenance of endothelial cell function [22]. FA was able to reduce blood pressure in hypertensive rats, and proved to be effective in improving endothelium-dependent relaxation in isolated thoracic aortic rings [23]. Furthermore, an endothelium-independent vascular relaxant effect of FA was also observed in different types of arteries (aortic, small mesenteric and coronary arteries), probably acting in calcium channel inhibition and calcium desensitization [24]. In addition, the ability of FA to reduce blood pressure in hypertensive mice is associated with nitric oxide (NO) -driven vasodilation and a reduction of NADPH-dependent superoxide anion levels [25,26]. In humans, the oral consumption of phenolic acids, including FA, improves blood flow and vascular responsiveness by inducing an acute change in endothelium-independent vasodilatation post consumption [27]. Moreover, small phenolics such as FA, have structural homologies to pharmacologic NADPH oxidase inhibitors, such as apocynin, and have been proposed as potent NAPDH oxidase inhibitors in endothelial cells [28]. These data suggest that consumption of phenolic acids may enhance vascular blood flow through maintenance of local NO levels [27]. Of this ability were phenolic metabolites, such as IFA, suggesting that they may be responsible for vascular activity [27]. NO is a key regulator of vasodilation, and it mediates a number of protective functions of the endothelium by inhibiting neutrophil activation and adhesion, platelet adhesion and aggregation, vascular smooth muscle proliferation and the expression of proinflammatory cytokines [29], and an imbalance in endothelium-derived NO can be a critical factor in the pathogenesis of vascular diseases, including hypertension, atherosclerosis and vasospasm with a decrease of blood flow [29]. In pathological conditions, NO bioavailability can be reduced by oxidation due to excessive production of superoxide anions in the vascular wall [30], suggesting that phenolic acids may have a role in the modulation of endothelial NO-dependent vasodilatation through antioxidant and redox-signaling mechanisms [31]. Moreover, eNOS uncoupling may have major consequences on endothelial function, and therefore enhancing eNOS activity by pre- and post-translational mechanisms (e.g., activation of the phosphatidylinositol 3-kinase/Akt pathway) is considered a pharmacological approach to maintain or restore the vascular function [32]. Limited evidence suggests that small phenolic acids such as FA can interact with endothelial cell signaling (notably PI3 kinase/Akt) leading to an increase in endothelial NO synthase (eNOS) activity and thus further NO production [33]; thus, the full mechanisms involved in phenolic acids modulation of NO balance are still poorly studied. Furthermore, although FA metabolites and AVN can be found in blood vessels at relevant concentrations [18,19], their effects have not yet been studied in depth. The present investigation aims to elucidate the molecular pathways involved in the modulation of NO balance in Primary Human Umbilical Vein Endothelial Cells (HUVEC) exposed to AVN-A, AVN-B, AVN-C, FA, IFA, DHFA and their metabolites FA glucuronide (FAG), DHFA glucuronide (DHFAG) and IFA sulfate (IFAS), at 1 µM, which is within a physiologically relevant concentration range [17,34]. NO levels, cyclic GMP (cGMP) levels, superoxide production and Akt1/eNOS activation will be assessed.

## 2. Materials and Methods

### 2.1. Chemicals

L-arginine (cod. A8094), apocynin (cod. 73536), sodium nitrite (cod. S2252), sodium nitrate (cod. S5506), Bradford reagent (cod. B6916), cytochrome c from bovine heart (C2037), sodium dodecyl sulfate (cod. 436143), superoxide dismutase from bovine erythrocytes (cod. S5395), L-NG-Nitro-Arginine (L-NNA, cod. N5501), phosSTOP (cod. PHOSS-RO), cOmpleteMini (cod. 04693124001), AVN-A (cod. 30366), AVN-B (cod. 93105), AVN-C (cod. 36465), FA (cod. 46278), IFA (cod. 05407) and DHFA (cod. 17803) were purchased by Sigma Aldrich (Gillingham, UK). FAG (cod. F308910), IFAS (cod. I816160) and DHFAG (cod. D448910) were obtained from Toronto Research Chemicals Inc (North York, Canada).

### 2.2. Materials for Cell Culture

The HUVEC cell line (Human Umbilical Vein Endothelial Cells, pooled donor, cod. C2519A), the medium EBM-2 with (cod. CC-3156) or without phenol red (cod. CC-3129), the BulletKit™—basal medium and SingleQuots™ (cod. CC-3156 and CC-4176) and ReagentPack Subculture Reagents (cod. CC-5034) with Trypsin/EDTA, TNS (Trypsin Neutralizer solution) and HEPES (4-(2-hydroxyethyl)1-iperazineethane sulfonic acid) solutions were obtained from Lonza (Basel, Switzerland).

### 2.3. Cell Culture

The HUVEC cells were used at passages 2–4 and were grown at 37 °C in a 5% CO_2_ humidified atmosphere. Cells were grown in T-75 flasks and subcultures were prepared using a trypsin/EDTA solution, and then seeded into 6-well or 96 well plates to be used for experiments. Growth medium was replaced the day after seeding, and every 2–3 days afterwards.

### 2.4. Cell Seeding and Exposures

HUVEC cells were seeded in 96-well plates (5 × 10^4^/mL; 100 µL/well; MTT assay, DAF2-DA assay) or 6 well plates (5 × 10^4^/mL; 2 mL/well; cGMP assay, Superoxide assay, protein detection) and grown to sub-confluence. For the MTT assay, cells were exposed to a range of concentrations of the compounds (0.1–10 µM, in serum free medium), or an equivalent volume of vehicle (MeOH) for the controls (0 µM) and incubated for 24 h. For all other assays, HUVEC cells were exposed to apocynin (100 µM), L-NNA (100 µM), the tested compounds (1 µM) or their vehicle as control (MeOH) in serum-free and phenol-red free medium containing L-arginin and incubated for 24 h.

### 2.5. MTT Assay

The MTT assay [35] was performed as follows: after the treatments (see Section 2.4), the medium was replaced by 100 µL of MTT solution (0.5 mg/mL in HBSS) and left for 4 h at 37 °C. The medium was removed, 100 µL of DMSO were added in each well and the absorbance was read at 570 nm by using a Multiskan Ex microplate reader (Thermo Fisher Scientific, Paisley, UK). After subtracting the blank values, data were converted to % of cells viability as follows: % cell viability = Abs sample/Abs control × 100.

### 2.6. LDH Assay

The lactate dehydrogenase (LDH) assay [36] was performed as follows: HUVECs (5 × 10^4^ cells/mL of medium) were seeded in 96-well plates (100 μL each well) and treated with the oat phenolics and metabolites (1 µM), apocynin (100 µM) or L-NNA (100 µM) for 24 h. Cytotoxicity was then determined by LDH assay kit by fol-lowing manufacturer’s instruction (Abcam, Cambridge, UK). Briefly, cell culture supernatants (10 μL) were collected from each well and were incubated with an LDH re-action mix for 30 min at 25 °C. LDH activity was then quantified by plate reader spec-trophotometric analysis at 450 nm and results were reported as % of the control values.

### 2.7. NO Analysis by DAF2-DA Fluorescence

The NO analysis was performed in exposed cells (see Section 2.4) using the DAF-2 diacetate (DAF2-DA) fluorescent probe [37] as follows: after the incubation time, a solution of DAF2-DA was added to the wells (0.5 µM final concentration, in HBSS) and left for 4 h at 37 °C. Basal fluorescence images were captured using a TIRF fluorescence microscopy system (Nikon, Minato City, Japan). The fluorescence signal was quantified on a FLx800 microplate reader (Biotek, Winooski, VT, USA) at excitation and emission wavelengths of 485 and 528 nm respectively. The fluorescence intensities were corrected by subtracting the non-specific fluorescence in wells without addition of DAF-2DA and in wells without cells.

### 2.8. Cyclic GMP Assay

In order to evaluate the cGMP production [38] in HUVEC cells exposed to tested compounds (see Section 2.4), the cGMP content was measured by using the Cyclic GMP competitive ELISA Kit (Cayman Chemical, Ann Arbor, MI, USA) following the manufacturer protocol.

### 2.9. Superoxide Production

The evaluation of superoxide production was performed by the ferricytochrome c reduction assay [39] as follows: HUVEC cells were treated with the compounds of interest (see Section 2.4), then washed with 50 mM phosphate buffer, pH 7.4, and exposed to ferricytochrome c (40 µmol/L) in HEPES-buffered isotonic salt medium. Reduction of ferricytochrome c in the supernatant was quantified at 550 nm (e = 21.1 mM^−1^ cm^−1^). Specificity of the assay for superoxide was assessed by superoxide dismutase co-incubation (SOD; 200 U/mL). Superoxide release was calculated from the difference in the setups without and with SOD and expressed as % of control.

### 2.10. Detection of Akt1 and eNOS Proteins by SDS-Page and Western Blotting

Treated cells were washed with ice-cold PBS and detached by using a cell scraper in lysis buffer (CelLytic M, Sigma, Gillingham, UK).) supplemented with phosphatase inhibitors (PhosSTOP, Sigma, Gillingham, UK) and protease inhibitors (cOmplete™ Mini, Sigma, Gillingham, UK). Cell lysates were centrifuged (12,500× *g*, 7 min, 4 °C), then supernatants containing the proteins were collected for the Western Blotting analysis. To determine the total protein concentration, 5 µL of the supernatants was aliquoted to be analyzed with the Bradford assay [40]. Ten micrograms of reduced and denatured proteins were separated in a Mini-PROTEAN^®^ Tetra system (140 V, 1 h) (Bio-Rad Laboratories Ltd., Watford, UK) by SDS-page on 9% (Akt1) or 6% (eNOS) polyacrylamide gels. Proteins were transferred onto nitrocellulose membranes (Santa Cruz Biotechnology, Dallas, TX, USA) in a Mini Trans-Blot module (40 V, 2 h) (Bio-Rad Laboratories Ltd., Watford, UK) and membranes were blocked with TTBS (Tris/HCl, pH 7.5, 100 mM NaCl, 0.1% Tween 20) containing 5% BSA for 30 min at room temperature. Excess BSA was removed by washing twice with TTBS for 5 min. Total eNOS (mouse monoclonal anti-NOS3, A-9, cod. sc-376751, final concentration 1:500), p-Akt1 (mouse monoclonal anti-p-Akt1, Ser 473, cod. sc-293125, final concentration, 1:500), Akt1 (mouse monoclonal anti-Akt1, B-1, cod. sc-5298, final concentration 1:500) and β-Actin (mouse monoclonal anti-β-Actin, C4, cod. sc-47778, final concentration 1:500). All primary antibodies were from Santa Cruz Biotechnology (Dallas, TX, USA). All antibodies used were suitable for Western Blotting. Primary antibodies were added to the membranes in TTBS containing 1% BSA (dilution 1:1000) and kept overnight at 4 °C. Membranes were washed two times with TTBS before incubation with the corresponding secondary fluorescent antibody (800CW or 680FD) obtained from Li-Cor(Lincoln, NE, USA) diluted in TTBS containing 1% BSA for 1 at room temperature. The membrane was washed again twice with TTBS and once with TBS. The bands were visualized using the Odyssey^®^ Fc Imaging System (Li-Cor, Lincoln, NE, USA). Images were taken, processed and quantified using the Image Studio Software (Li-Cor, Lincoln, NE, USA).

### 2.11. Statistical Analysis

Data are expressed as means ± SEM. Statistical analysis was performed using the Graph Pad Prism version 7.0 software. MTT data were entered using a grouped analysis format and were analyzed by 2-way ANOVA followed by Tukey’s multiple comparisons test with a confidence level of 95%, to assess the effect of treatments at the stated concentrations. All other results were analyzed by one-way ANOVA followed by Tukey’s multiple comparisons test with a confidence level of 95%. Significance level was set at *p* < 0.05.

## 3. Results

### 3.1. Cytotoxicity of Test Compounds in HUVEC Cells

**Figure 1:**
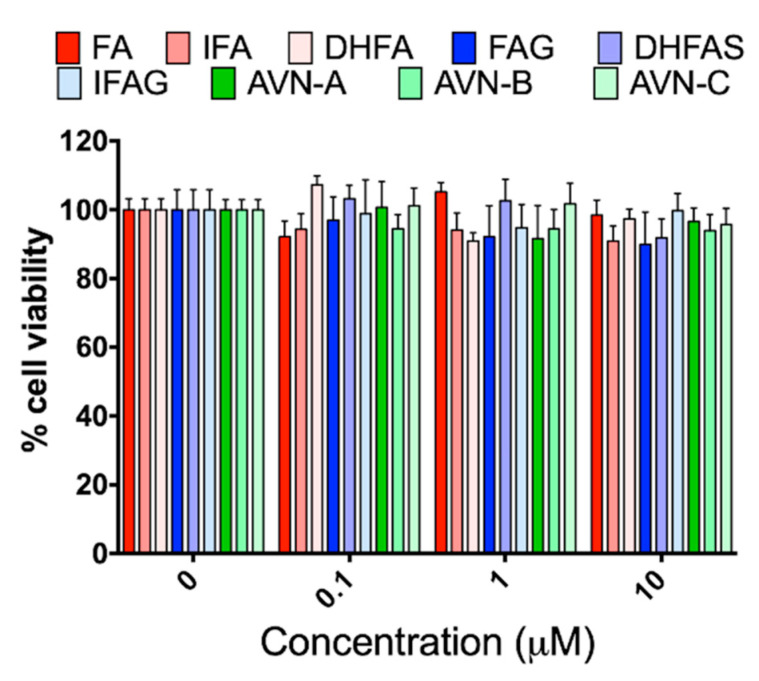
HUVEC cell viability, measured with the MTT assay, after pre-treatment with the oat phenolics and metabolites (0.1, 1 and 10 µM) ferulic acid (FA) isoferulic acid (IFA), dihydroferulic acid (DHFA), FA glucuronide (FAG), DHFA glucuronide (DHFAG), IFA sulfate (IFAS), avenanthramide A (AVN-A) avenanthramide B (AVN-B) avenanthramide C (AVN-C) for 24 h. Data are expressed as percentage of control values (100%) and reported as average ± SEM of 4 independent experiments performed in triplicate. *p* > 0.05 vs. control (*n* = 12).

Before investigating the effects of the test compounds on the NO system, HUVEC cells were exposed to different concentrations of the tested compounds (0.1, 1 and 10 µM) for 24 h to ascertain any potential cytotoxic activity through MTT assay. As shown in Figure 1, we did not observe any significant reduction of cell viability with any of the compounds, at all the concentrations tested (*p* > 0.05).

**Figure 2:**
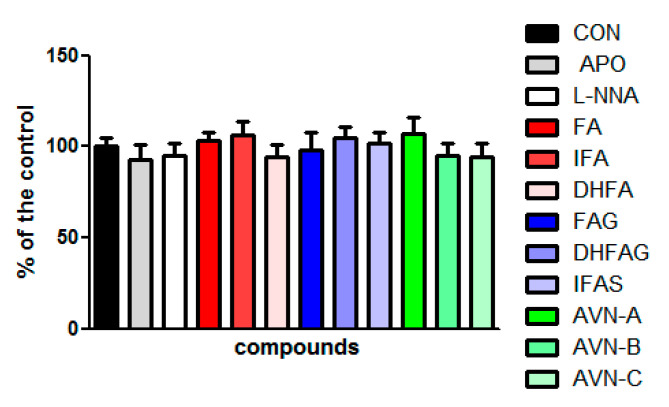
HUVEC cell viability measured with the LDH assay, after pre-treatment with the oat phenolics and metabolites (1 µM) ferulic acid (FA) isoferulic acid (IFA), dihydroferulic acid (DHFA), FA glucuronide (FAG), DHFA glucuronide (DHFAG), IFA sulfate (IFAS), avenanthramide A (AVN-A) avenanthramide B (AVN-B) avenanthramide C (AVN-C), apocynin (APO, 100 µM) or L-NG-Nitro-Arginine (L-NNA, 100 µM) for 24 h. Data are expressed as percentage of control (CON) values (100%) and reported as average ± SEM of 4 independent experiments performed in triplicate. *p* > 0.05 vs. control (*n* = 12).

Moreover, the LDH assay was carried out to further evaluate the potential cytotoxicity of the compounds at the concentration of 1 µM and of apocynin (100 µM) and L-NNA (100 µM). The LDH assay provides an assessment of cell damage and membrane integrity. The results show that none of the compounds tested affected the cell viability (*p* > 0.05) (Figure 2).

### 3.2. Modulation of Endothelial NO Levels by DAF2-DA Fluorescence

The DAF2-DA fluorescence probe was used to assess NO levels in HUVEC cells treated with the compounds of interest (1 µM). Apocynin (100 µM), which is an inhibitor of NADPH oxidase enzyme, and L-NNA (100 µM), a competitive inhibitor of NO synthase (NOS) derived from N(ω)-nitro-L-arginine methyl ester (L-NAME) hydrolysis, were also used as additional controls for all NO related assays. We assessed the cellular levels of NO in intact living HUVEC cells, measured as DAF2-DA fluorescence intensity, which is proportional to NO levels.

**Figure 3:**
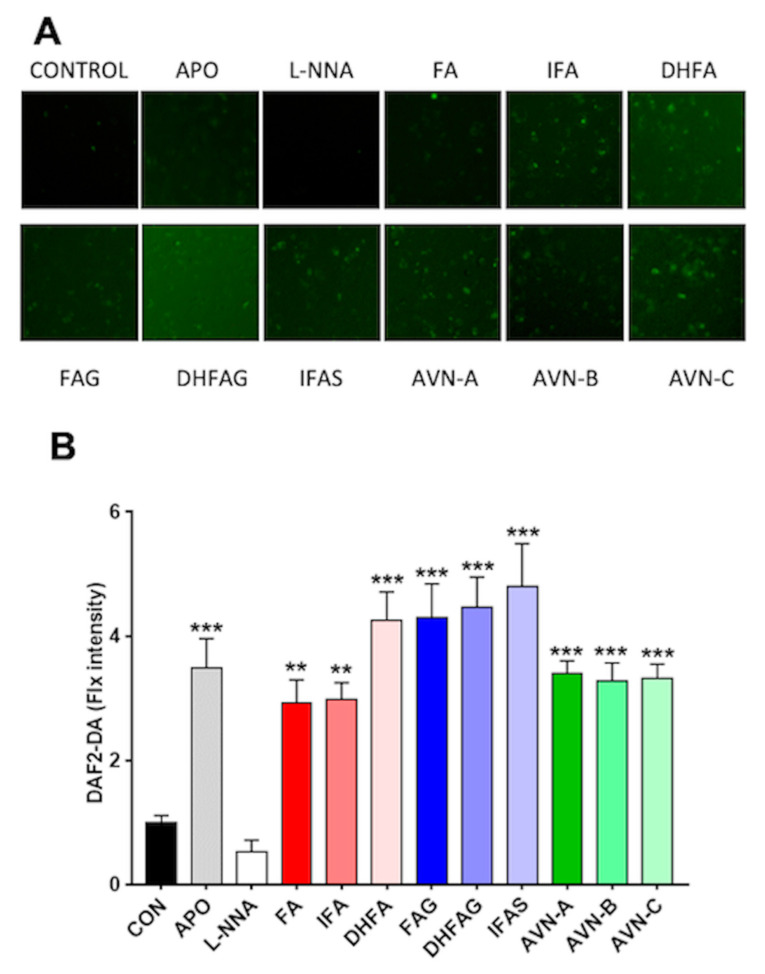
NO levels in HUVEC as visualized by DAF-2DA fluorescence (**A**) and relative fluorescence intensities (**B**), after pre-treatment with the oat phenolics and metabolites (1 µM), ferulic acid (FA) isoferulic acid (IFA), dihydroferulic acid (DHFA), FA glucuronide (FAG), DHFA glucuronide (DHFAG), IFA sulfate (IFAS), avenanthramide A (AVN-A) avenanthramide B (AVN-B) avenanthramide C (AVN-C), apocynin (APO, 100 µM) or L-NG-Nitro-Arginine (L-NNA, 100 µM) for 24 h. Images in panel A are a representative example, whereas data with error bars in panel B represent the average ± SEM of 6 independent experiments and are expressed as fold change (control, CON, =1). ** = *p* < 0.01, *** = *p* < 0.001 vs. control (*n* = 6).

Untreated cells (control) had a low fluorescence signal (Figure 3), which was further attenuated in the cells exposed to the NO synthase inhibitor L-NNA, whereas that cells treated with apocynin showed a significant increase in fluorescence intensity compared to the control (*p* < 0.05). Similar to the apocynin group, the cells exposure to all phenolic compounds tested significantly enhanced the fluorescence signal compared to the control group (*p* < 0.05), with the highest signal observed for IFAS (*p* < 0.001).

### 3.3. Modulation of cGMP Production by ELISA Assay

One of the major mechanism through which the effects of NO are mediated is the production of the second messenger cGMP [36]; therefore, we analyzed the cGMP levels in HUVEC cells as an indirect measure of NO release after treatments with the compounds of interest.

**Figure 4:**
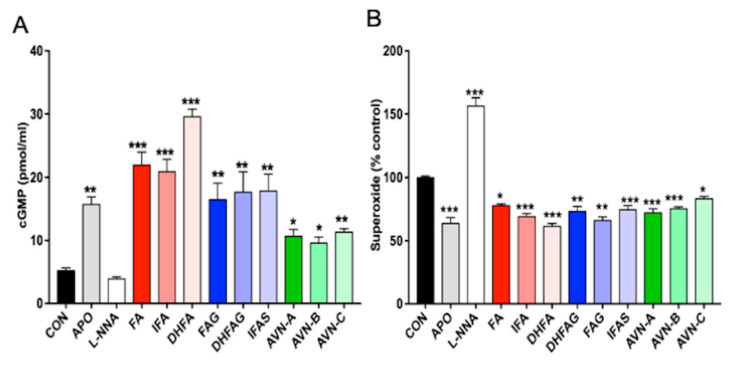
Cyclic GMP levels (**A**) and Superoxide levels (**B**) in HUVEC cells, measured after pre-treatment with the oat phenolics and metabolites (1 µM), ferulic acid (FA) isoferulic acid (IFA), dihydroferulic acid (DHFA), FA glucuronide (FAG), DHFA glucuronide (DHFAG), IFA sulfate (IFAS), avenanthramide A (AVN-A) avenanthramide B (AVN-B) avenanthramide C (AVN-C), apocynin (APO, 100 µM) or L-NG-Nitro-Arginine (L-NNA, 100 µM) or controls (CON, vehicle) for 24 h. Data with error bars represent the average ± SEM of 3 independent experiments performed in duplicated for cGMP, and of 4 independent experiments performed in triplicate for Superoxide. * = *p* < 0.05, ** = *p* < 0.01, *** = *p* < 0.001 vs. control (*n* = 6 for cGMP and *n* = 12 for superoxide).

As shown in Figure 4 (panel A), the pre-treatment with apocynin led to a significant increase in cGMP levels compared to the control group (*p* < 0.05), whereas cells exposed to the NO synthase inhibitor L-NNA were found to have level of cGMP comparable to control cells (*p* > 0.05). All compounds tested at the concentration of 1 µM led to a significant increase in the cGMP levels compared to control cells. DHFA, FA and IFA proved to be the most effective in enhancing cGMP release (*p* < 0.001), followed by IFAS, DHFAG, FAG and AVN-C (*p* < 0.01), AVN-A and AVN-B (*p* < 0.05).

### 3.4. Evaluation of the Superoxide Release from Intact Cells

Superoxide ions can easily react with NO and decrease its availability, thus in view of the antioxidant potential of the phenolic compounds and their metabolites, the assessment of superoxide levels is considered critical. The superoxide production was measured in HUVEC cells treated with the phenolic compounds for 24 h through analyzing ferricytochrome c reduction in presence or absence of superoxide dismutase (SOD). The results are expressed as percentage of superoxide production of the control cells (Figure 4, panel B). The pre-treatment with apocynin led to a significant decrease in superoxide levels compared to the control group (*p* < 0.001), whereas cells exposed to L-NNA were found to have significantly higher levels compared to control cells (*p* < 0.001). Cells pre-treated with all the phenolic compounds showed a significant decrease of superoxide release compared to control cells (*p* < 0.05). In particular, DHFA, IFA, DHFA-g, AVN-A and AVN-B were found to be the most effective (*p* < 0.001), to a level comparable to apocynin.

### 3.5. Akt1/eNOS Activation in Endothelial Cells

**Figure 5:**
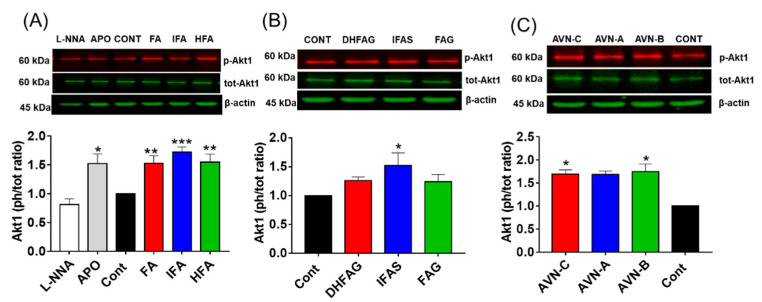
Akt1 activation state, measured as phosphorylation (Ser 473)/total ratio in HUVEC pre-treated for 2 h with the un-conjugated oat phenolics (**A**), sulfate and glucuronide metabolites (**B**) and avenanthramides (**C**) at physiologically relevant concentration (1 µM). Controls (CON) Ferulic acid (FA) isoferulic acid (IFA), dihydroferulic acid (DHFA), FA glucuronide (FAG), DHFA glucuronide (DHFAG), IFA sulfate (IFAS), avenanthramide A (AVN-A) avenanthramide B (AVN-B) avenanthramide C (AVN-C) were tested. Apocynin (APO, 100 µM) and L-NG-Nitro-Arginine (L-NNA, 100 µM) were used as additional controls (**A**). Data with error bars represent the average ± SEM of 6 independent experiments and are expressed as phospho-Akt1/ total-Akt1 ratio. * = *p* < 0.05, ** = *p* < 0.01, *** = *p* < 0.001 vs. control (*n* = 6).

To investigate the role of the Akt/eNOS pathway in the maintenance of NO balance, we initially determined the activation state of Akt1 in HUVEC cells pre-treated with the compounds through western immunoblotting. The protein band intensity values were normalized using the corresponding values of β-actin and the activation state of Akt1 was expressed as a phosphorylated/total protein ratio. Results (Figure 5) showed that the activation state of Akt1 (phospho/total ratio) was significantly increased when cells were pre-treated with apocynin (*p* < 0.05), and not significantly altered by L-NNA (*p* > 0.05).

Similar to apocynin, the phenolic aglycones (Figure 5A), sulfate metabolite (Figure 5B) and AVN (Figure 5C) were able to significantly increase Akt1 activation (*p* < 0.05), whereas the glucuronides (Figure 5B) did not (*p* > 0.05).

**Figure 6:**
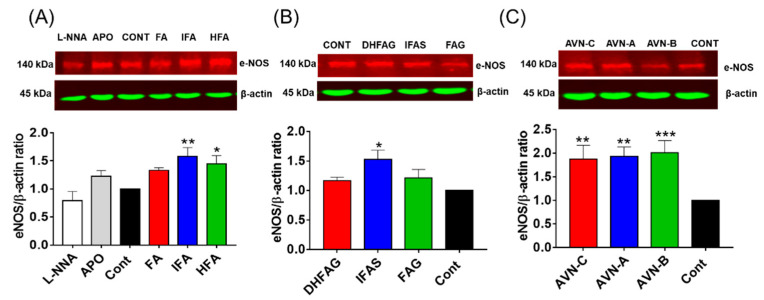
eNOS total protein levels measured in HUVEC pre-treated for 24 h with the un-conjugated oat phenolics (**A**), sulfate and glucuronide metabolites (**B**) and avenanthramides (**C**) at physiologically relevant concentration (1 µM). Controls (CON) Ferulic acid (FA) isoferulic acid (IFA), dihydroferulic acid (DHFA), FA glucuronide (FAG), DHFA glucuronide (DHFAG), IFA sulfate (IFAS), avenanthramide A (AVN-A) avenanthramide B (AVN-B) avenanthramide C (AVN-C) were tested. Apocynin (APO, 100 µM) and L-NG-Nitro-Arginine (L-NNA, 100 µM) Apocynin (100 µM) and L-NNA (100 µM) were used as additional controls (**A**). Data with error bars represent the average ± SEM of 6 independent experiments and are expressed as phospho-Akt1/ total-Akt1 ratio. Protein levels were normalized against β-actin levels. * = *p* < 0.05, ** = *p* < 0.01, *** = *p* < 0.001 vs. control (*n* = 6).

eNOS expression was also assessed through western Immunoblotting, and the protein band intensity values were normalized using the corresponding values of β-actin. The expression of eNOS (Figure 6) was significantly increased (*p* < 0.05) in cells pre-treated with the IFA and DHFA (Figure 6A), IFAS (Figure 6B) and AVN (Figure 6C).

## 4. Discussion

The present study aimed to explore the ability of AVNs, FA, IFA and HFA, in comparison to their glucuronide and sulfate metabolites, to influence NO balance at endothelial levels. These compounds were tested in HUVEC cell model that simulated the vessel endothelium, at 1 μM concentration, which, based on bioavailability data from randomized controlled trials, is a physiologically relevant concentration range in the bloodstream after ingestion of foods containing the parent phenolic compounds [17,41]. After treatments with the compounds tested, endothelial NO levels were assessed using the NO-sensitive phluorophore DAF2-DA in intact living HUVEC cells: a significantly stronger signal was observed in presence of the known NADPH oxidase inhibitor apocynin, as well as all phenolic compounds and metabolites tested. NO is a known regulator of soluble guanylate cyclase (sGC), which converts GTP to the intracellular signaling molecule cGMP, a molecule which is responsible for many of the physiological effects of NO [42,43]. Therefore, we assessed the cellular cGMP levels in HUVEC cells pre-treated with the phenolic compounds and metabolites. As expected, cGMP levels were also significantly increased, and matched the NO increased levels observed through DAF-2DA reactivity. Overall, our data obtained through DAF-2DA imaging and cGMP levels shows a significantly higher cellular steady-state level of NO, which can be the result of two opposing processes, an enhanced NO production and/or a reduced NO degradation [42], and both mechanism were considered in our study design. Superoxide production can occur in endothelial cells through the action of NADPH oxidases [44]: superoxide radicals decrease NO level through the production of peroxynitrite [45], therefore superoxide formation in endothelial cells is intimately connected to NO balance within the cells [44]. In our experiments, superoxide levels were quantified by one-electron oxidation with ferricytochrome c within the cells, in presence or absence of superoxide dismutase, to assess the specificity of the assay to superoxide. All the compounds proved to be effective and significantly reduced superoxide levels in the cells. The tested phenolics could have been able to reduce cellular superoxide levels in different ways: through direct scavenging activity, by acting as primary antioxidants, or acting as NADPH oxidase inhibitors. For example, Steffen et al. showed that (-)-epicatechin, a flavan-3-ol, was effective in inhibiting superoxide production working as a primary antioxidant but not as NAPDH oxidase inhibitor, whereas the metabolites 3′- and 4′-*O*-methyl epicatechin were acting as NADPH oxidase inhibitors [39]. Similar to methylated forms of epicatechin, FA and other phenolics were also able to exert apocynin-like activity [46]. Our results are in agreement with the study by Suzuki et al., where FA was shown to increase NO bioavailability through a reduction of NADPH-dependent superoxide anion levels in rat aortas [25]. The positive modulation of NO balance occurs through eNOS modulation and can be enhanced by different stimuli and intracellular signals; one of the key signals involved is the phosphorylation of the PI3 kinase Akt [47]. Different phenolic compounds have already been shown to induce Akt phosphorylation in endothelial cells [48,49]. Most compounds tested in this study, with the exception of the glucuronides, significantly activated Akt and increased eNOS expression. IFA showed the best activity, and its sulfate was the only conjugate metabolite to significantly increase eNOS levels. Interestingly, other studies showed a higher effect of sulfate metabolites compared to the parent aglycone; for example, it was observed that FA-4-*O*-sulfate is more active than FA in lowering blood pressure in mice [50]. Similarly, metabolites of other phenolic acids (e.g., hydroxytyrosol and tyrosol) are shown to exert significant biological activities comparable to or higher than their parent compounds [51,52,53,54,55]. It is suggested that the different effectiveness of sulfated metabolites compared to glucuronides can in some cases be related to differences in cellular uptake and cell internalization [55]. Thus, conjugated forms are not always more effective than their parental forms: for example, resveratrol was able to elevate eNOS enzyme activity and endothelial NO release whereas its glucuronide and sulfate metabolites did not show any significant effect [56]. In our study, the sulfated metabolite has proven to be more effective than the parental aglycone form. We evaluated one of the possible pathways involved in the regulation of the NO balance, but it cannot be excluded that other intracellular signals are modulated by the tested compounds at the same time. Likewise, further studies will be needed to define the role of these compounds in the eNOS downstream signaling pathways, which may influence the endothelial cells homeostasis by increasing the mitochondrial biogenesis and through the activation of mTOR and the expression of SIRT1 [57,58,59,60]. Overall, our mechanistic investigation explores some vascular mechanisms that complement in vivo investigations [61], providing additional evidence that phenolic acids derived from oats and their metabolites could contribute to the cardiovascular benefits derived from oat consumption.

## 5. Conclusions

**Figure 7:**
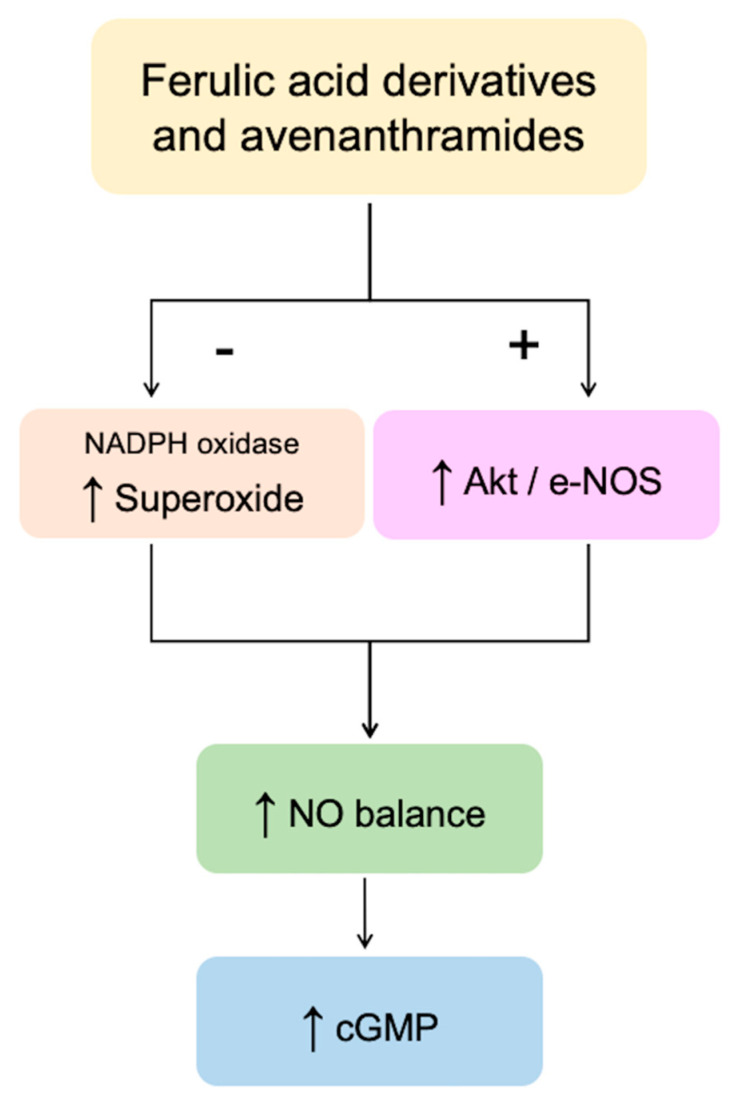
Schematic representation of the mechanisms involved in the modulation of endothelial NO levels by the oat phenolics and metabolites tested in the present study. + = activation; − = inhibition; ↑ = increased.

In summary, our results indicated that bioavailable oat phenolics and their circulating conjugated metabolites, when tested at physiologically relevant concentrations, are able to modulate NO balance in HUVEC cells (Figure 7).

All compounds tested were able to decrease NO degradation (via reduced superoxide formation); however, the glucuronide-conjugated metabolites are not able to significantly enhance NO production through the Akt1/eNOS pathway. Our observations further confirm that metabolic conversion of phenolics forms found in the food matrix into metabolic products does not necessarily compromise their bioactivity.

## Abbreviations

eNOS	endothelial nitric oxide synthase
L-NNA	*N*-γ-nitro-l-arginine methyl ester
NO	nitric oxide
cGMP	cyclic guanosine monophosphate
FA	ferulic acid
IFA	isoferulic acid
HFA	hydroferulic acid
FAG	ferulic acid 4-*O*-glucuronide
DHFAG	dihydro ferulic acid 4-*O*-β-d-glucuronide
IFAS	isoferulic acid 3-*O*-sulfate
AVN-A	avenanthramide A
AVN-B	avenanthramide B
AVN-C	avenanthramide C
SOD	superoxide dismutase
